# Large Pneumoperitoneum After Gastrostomy Placement in a Patient With Amyotrophic Lateral Sclerosis

**DOI:** 10.7759/cureus.37298

**Published:** 2023-04-08

**Authors:** Javier Navarro-Esteva, Marta Evora-Garcia, Guillermo Perez-Mendoza

**Affiliations:** 1 Pulmonary Medicine, Hospital Universitario Gran Canaria Dr. Negrín, Las Palmas de GC, ESP

**Keywords:** acute respiratory failure (arf), massive pneumoperitoneum, neuromuscular diseases, noninvasive positive pressure ventilation, percutaneous gastrostomy

## Abstract

We present a 67-year-old male with amyotrophic lateral sclerosis (ALS) who developed left lower lobe atelectasis and respiratory failure caused by a large pneumoperitoneum after gastrostomy placement. The patient was successfully managed with paracentesis, postural measures, and continued application of noninvasive positive pressure ventilation (NIPPV). There is no clear evidence that links the use of NIPPV with an increased risk of pneumoperitoneum. The evacuation of air from the peritoneal cavity may help improve the respiratory mechanics in patients with diaphragmatic weakness such as the one presented.

## Introduction

Patients with amyotrophic lateral sclerosis (ALS) may need a percutaneous gastrostomy to ensure adequate nutrition. This procedure requires the adoption of the supine posture and insufflation of air into the stomach, which may restrict the diaphragmatic excursion causing discomfort and increasing the risk of respiratory failure. However, the gastrostomy can be carried out while the patient is on noninvasive positive pressure ventilation (NIPPV), which may improve the respiratory mechanics and avert such risk. Concerning complications, the finding of a pneumoperitoneum is not a rare one, but it is usually self-limited and does not usually preclude its use for feeding. Therefore, it is often referred to as "benign pneumoperitoneum". The question of whether NIPPV may increase the risk of developing a pneumoperitoneum has been looked at by previous authors [1-3]. To the best of our knowledge, there are no reports regarding paracentesis as a procedure to evacuate intraabdominal air to improve respiratory mechanics in patients with diaphragmatic weakness such as those with ALS.

## Case presentation

**Figure 1:**
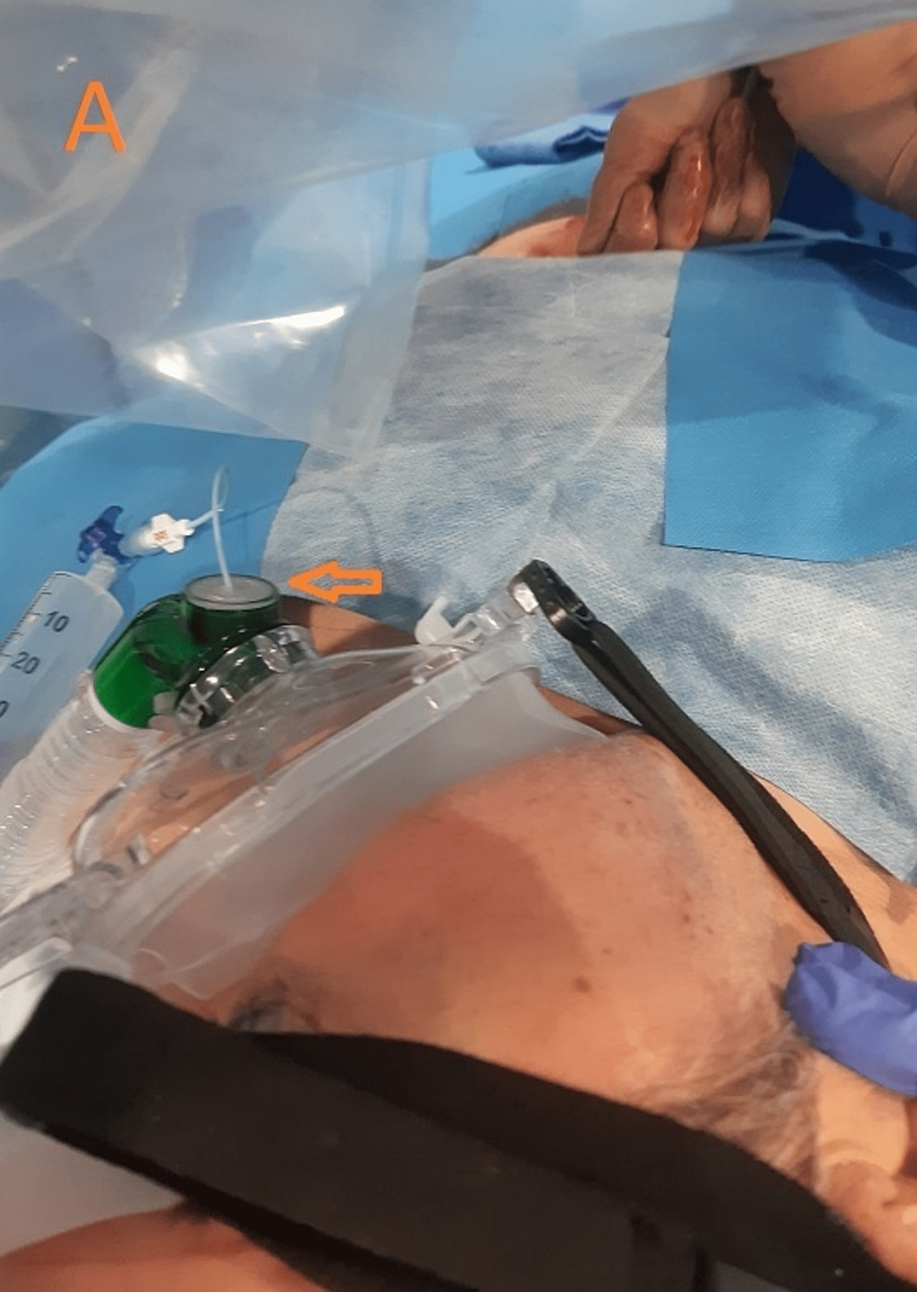
Preparation for gastrostomy placement under noninvasive positive pressure ventilation (NIPPV). The use of an adaptor (arrow) allows passage of a nasogastric tube and execution of the procedure under NIPPV.

**Figure 2:**
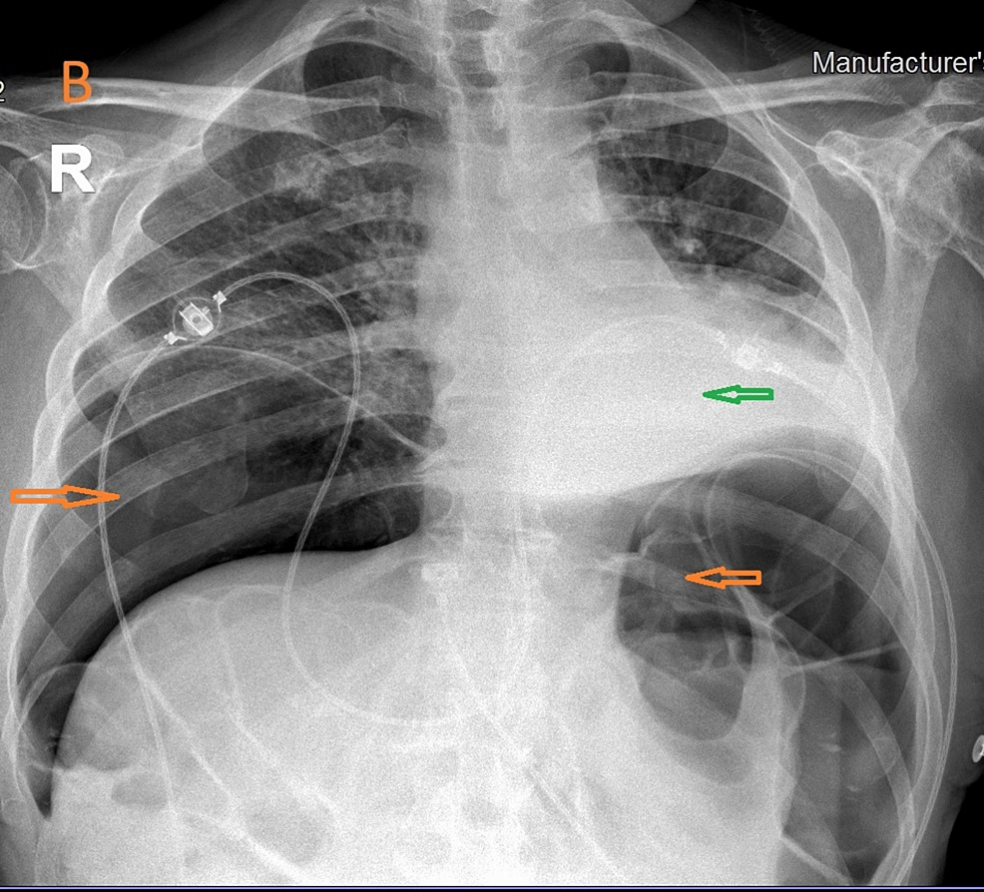
Upright Chest X-ray two days after the procedure. A large pneumoperitoneum (orange arrows) and collapse of the left lower lobe (green arrow) are observed.

**Figure 3:**
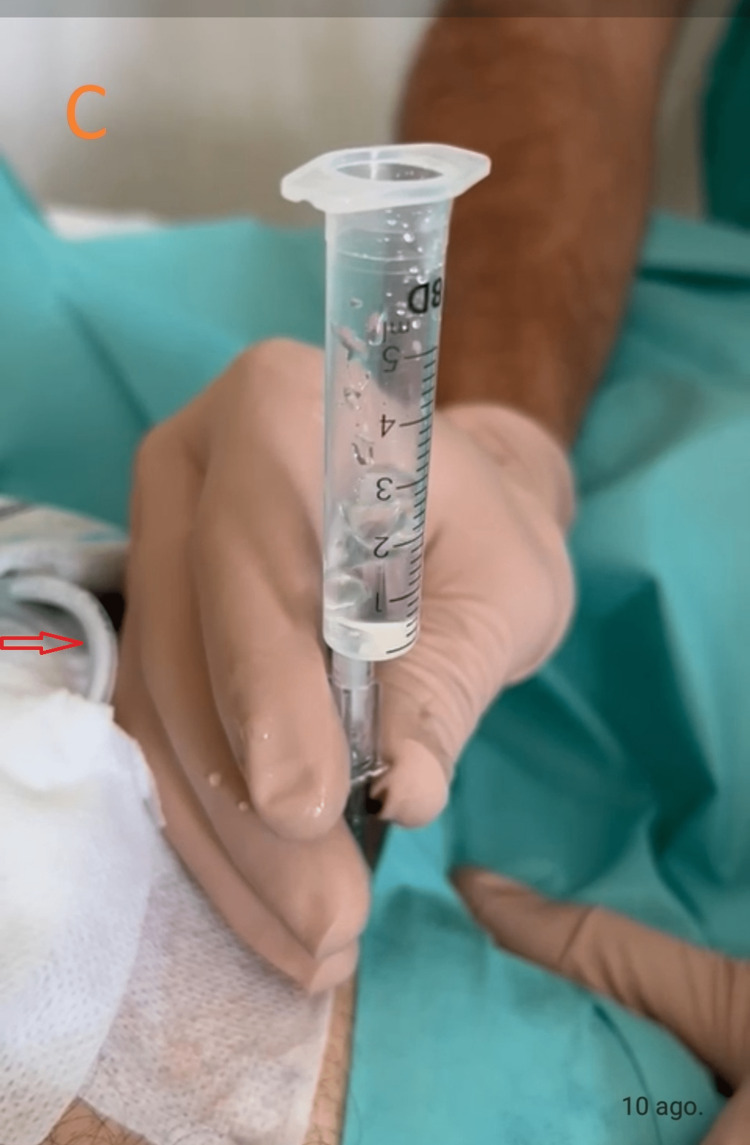
Paracentesis. A 16-gauge cannula was inserted into the left upper quadrant, lateral to the rectus muscle. With the aid of water in the syringe, spontaneous airflow is noted. The gastrostomy tube is shown (arrow).

**Figure 4:**
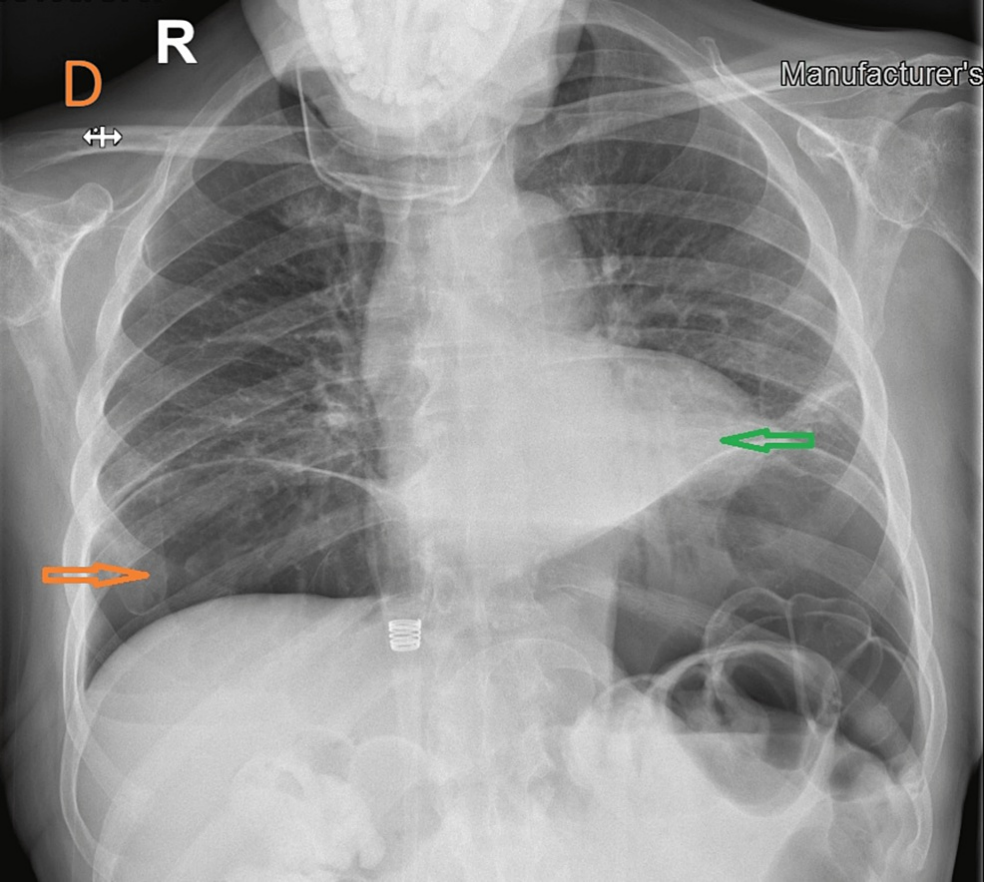
Upright Chest X-ray after paracentesis. Reduction in the size of the pneumoperitoneum and partial resolution of lobar collapse is observed.

**Figure 5:**
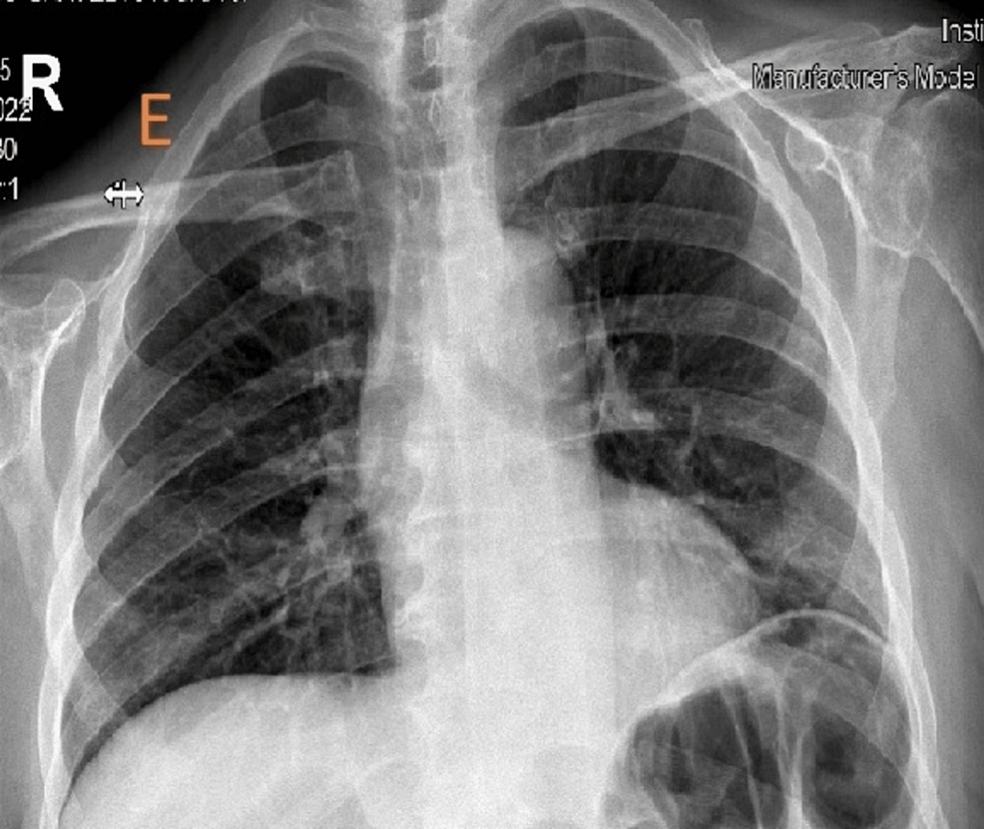
Upright Chest X-ray after two months. Complete resolution of the pneumoperitoneum.

A 67-year-old male with ALS and moderate bulbar dysfunction was compliant with nocturnal NIPPV (oronasal mask, S/T mode, inspiratory positive airway pressure [IPAP] 16 cm H2O, expiratory positive airway pressure [EPAP] 4 cm H2O, back-up rate 14 breaths per minute, medium-rise time, minimum and maximum inspiratory time 1 and 2 seconds respectively, expiratory trigger at 25% of peak expiratory flow), and was scheduled for percutaneous radiologic gastrostomy. He could not perform spirometry properly and did not require additional oxygen to the mechanical ventilation. The procedure was performed under NIPPV (Figure 1) because it felt safer for the patient, who would otherwise complain of orthopnea. Two days after the uneventful gastrostomy, the patient reported dyspnea, and left-sided trepopnea, and was found in respiratory failure (on venturi mask fraction of inspired oxygen [FiO2] 0.4, arterial pH 7.41, partial pressure of carbon dioxide [PaCO2] 50 mm Hg, partial pressure of oxygen [PaO2] 63 mm Hg, bicarbonate [HCO3] 29 mmol/L). The newly placed tube had not been used for any purpose. The absence of fever, abdominal bloating, or tenderness did not raise suspicion of a ruptured viscus. Likewise, signs of hemodynamic instability such as tachycardia or low blood pressure were not observed. However, on chest X-ray, a large pneumoperitoneum and collapse of the left lower lobe were noted (Figure 2). The surgical team was consulted and decided not to operate. His dyspnea and hypoxemia improved by adopting the right lateral decubitus but, given his condition and after consulting with gastroenterology, paracentesis was attempted. Just before needle decompression, the patient was placed in the supine position for ultrasonography, which confirmed the presence of air in the peritoneum; the stratosphere sign was observed. Immediately afterward, the patient reported further relief from the shortness of breath (Figures 3, 4, and Video 1 in Appendix). Then, parenteral fluids, omeprazole, and an antibiotic were administered. Forty-eight hours later, the gastrostomy tube was used for nutrition without complications. Finally, the patient was discharged home, and two months after he reported doing well, with complete resolution of the pneumoperitoneum (Figure 5).

## Discussion

Pneumoperitoneum is not a rare complication of gastrostomy, although it is usually non-massive and self-limited [1-5]. In a large case series, 33 out of 722 patients were diagnosed with pneumoperitoneum, but none required surgery [4]. Gastric insufflation is required before puncturing the stomach; therefore, it is a source of air that may leak into the abdominal cavity. Hence, the incidence of pneumoperitoneum has been reported regardless of NIPPV use during the procedure [4,5]. The lower esophageal sphincter pressure of patients with ALS does not seem to differ from healthy subjects [6]. However, if NIPPV exceeded the sphincter pressure, then NIPPV would as well have contributed to the pneumoperitoneum. However, the level of the airway pressure that was applied and the absence of prior abdominal bloating makes this hypothesis questionable. Other authors indicate that gastrostomies are currently being placed in NIPPV-dependent ALS patients submitted to airway pressures of up to 25 cm H2O or to exhaled tidal volumes higher than 800 ml during the procedure, without significant complications being reported [1,2]. A similar concern has been raised when NIPPV is used immediately after bariatric surgery. However, a recent systematic review does not provide evidence of a signal that there is an increased anastomotic dehiscence risk when NIPPV is used in such a scenario [7]. In our case, once paracentesis was done, NIPPV was continued during sleep at a slightly lower IPAP level without recurrence of symptoms. Mechanical cough assistance had not been employed during this timeframe. Accumulation of peritoneal air is also reported after procedures such as esophageal stent placement or endoscopic submucosal dissection of colonic tumors, where successful drainage has been communicated [8,9].

## Conclusions

In conclusion, this is a patient with impaired diaphragmatic strength whose respiratory function was further compromised by a large pneumoperitoneum, who benefited from drainage by possibly improving his respiratory system compliance. We do not find scientific evidence indicating that NIPPV is associated with an increased risk of pneumoperitoneum during gastrostomy placement. NIPPV may relieve dyspnea and avoid respiratory failure by improving the patient´s respiratory mechanics.
